# Supplementary figures and images for: The motivation for physical activity is a predictor of VO2peak and is a useful parameter when determining the need for cardiac rehabilitation in an elderly cardiac population
Source: PLoS One. 2022 Sep 28;17(9):e0275091. doi: 10.1371/journal.pone.0275091 (PMC9518852; doi:10.1371/journal.pone.0275091)

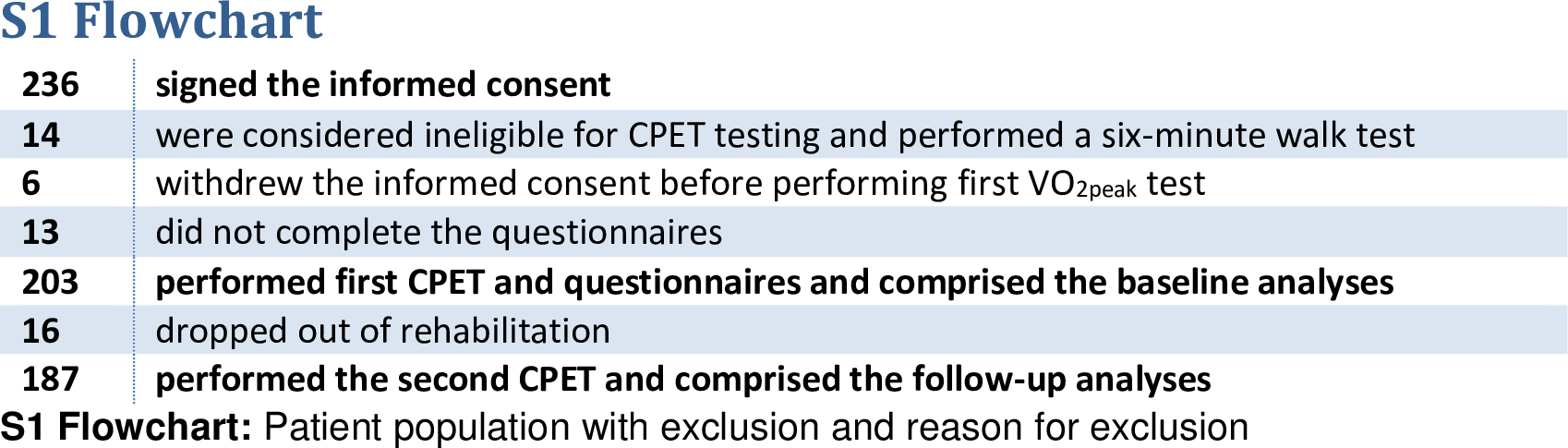

Supplement: S1 Flowchart — (TIF) [file pone.0275091.s001.tif]

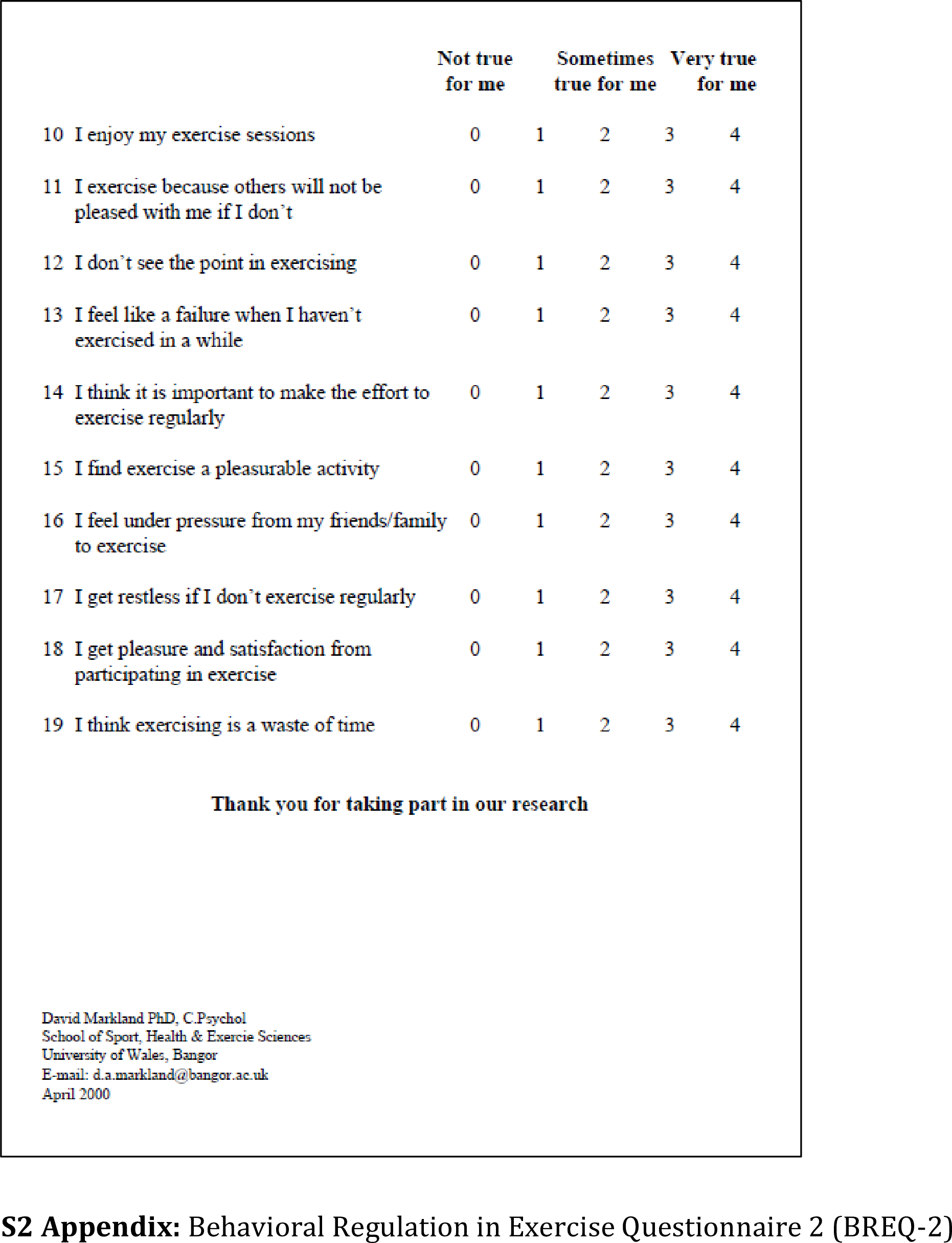

Supplement: S1 Appendix — (ZIP) [file pone.0275091.s002.zip › S1b_Appendix.tif]

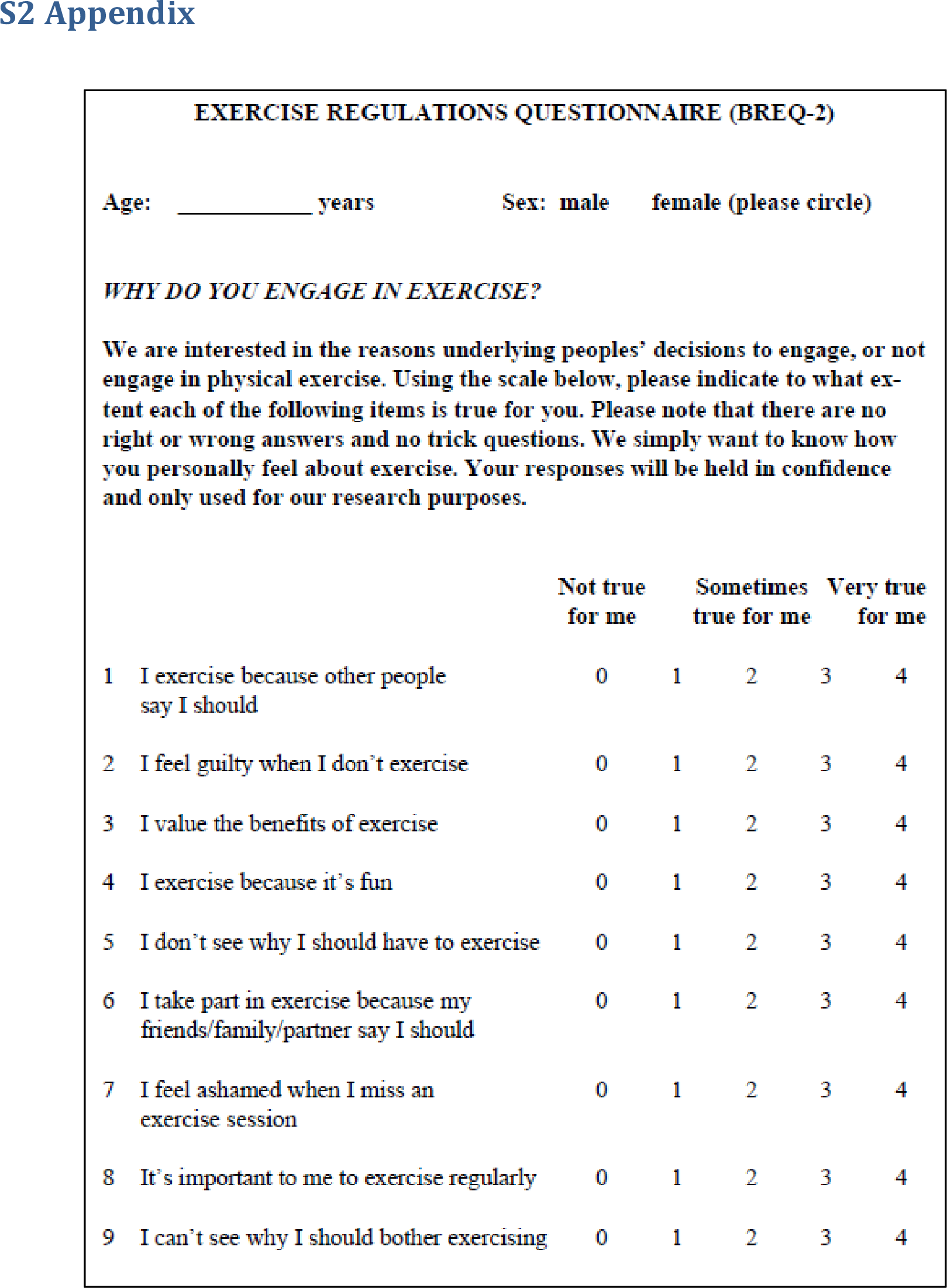

Supplement: S1 Appendix — (ZIP) [file pone.0275091.s002.zip › S1a_Appendix.tif]
