# Supplementary material for: The motivation for physical activity is a predictor of VO2peak and is a useful parameter when determining the need for cardiac rehabilitation in an elderly cardiac population
Source: PLoS One. 2022 Sep 28;17(9):e0275091. doi: 10.1371/journal.pone.0275091 (PMC9518852; doi:10.1371/journal.pone.0275091)
Supplement: Attachment — Submitted filename: Response to reviewers.docx [file pone.0275091.s003.docx]

August 30, 2022

**Resubmission of manuscript:** *The motivation for physical activity is a predictor of VO_2peak_ and is a useful parameter when determining the need for cardiac rehabilitation in an elderly cardiac population*

Dear Editor and reviewers

Thank you again for reconsidering our revised article “*The motivation for physical activity is a predictor of VO_2peak_ and is a useful parameter when determining the need for cardiac rehabilitation in an elderly cardiac population*”.

We have addressed the comments and questions in this response below. Our responses to the reviewers’ comments are marked yellow and a manuscript with tracked changes has been added to the submission, which addresses the comments.

Sincerely, on behalf of the authors

Nicolai Mikkelsen

PONE-D-21-02329R2

The motivation for physical activity is a predictor of VO2peak and is a useful parameter when determining the need for cardiac rehabilitation in an elderly cardiac population

PLOS ONE

Dear Dr. Mikkelsen,

Thank you for submitting your manuscript to PLOS ONE. After careful consideration, we feel that it has merit but does not fully meet PLOS ONE’s publication criteria as it currently stands. Therefore, we invite you to submit a revised version of the manuscript that addresses the points raised during the review process.

==============================

Dear authors, Thank you for this revised manuscript. I agree with the reviewers that the comments of the previous reviewers have been sufficiently taken into account. Some minor issues in particular on the characteristics of the population studied still need to be addressed (see 6. Review Comments to the Author). I am sure you will be able to deal with them easily.

==============================

Please submit your revised manuscript by Sep 08 2022 11:59PM. If you will need more time than this to complete your revisions, please reply to this message or contact the journal office at plosone@plos.org. When you're ready to submit your revision, log on to https://www.editorialmanager.com/pone/ and select the 'Submissions Needing Revision' folder to locate your manuscript file.

Please include the following items when submitting your revised manuscript:

A rebuttal letter that responds to each point raised by the academic editor and reviewer(s). You should upload this letter as a separate file labeled 'Response to Reviewers'.

A marked-up copy of your manuscript that highlights changes made to the original version. You should upload this as a separate file labeled 'Revised Manuscript with Track Changes'.

An unmarked version of your revised paper without tracked changes. You should upload this as a separate file labeled 'Manuscript'.

If you would like to make changes to your financial disclosure, please include your updated statement in your cover letter. Guidelines for resubmitting your figure files are available below the reviewer comments at the end of this letter.

If applicable, we recommend that you deposit your laboratory protocols in protocols.io to enhance the reproducibility of your results. Protocols.io assigns your protocol its own identifier (DOI) so that it can be cited independently in the future. For instructions see: https://journals.plos.org/plosone/s/submission-guidelines#loc-laboratory-protocols. Additionally, PLOS ONE offers an option for publishing peer-reviewed Lab Protocol articles, which describe protocols hosted on protocols.io. Read more information on sharing protocols at https://plos.org/protocols?utm_medium=editorial-email&utm_source=authorletters&utm_campaign=protocols.

We look forward to receiving your revised manuscript.

Kind regards,

Annett Salzwedel

Academic Editor

PLOS ONE

Journal Requirements:

Please review your reference list to ensure that it is complete and correct. If you have cited papers that have been retracted, please include the rationale for doing so in the manuscript text, or remove these references and replace them with relevant current references. Any changes to the reference list should be mentioned in the rebuttal letter that accompanies your revised manuscript. If you need to cite a retracted article, indicate the article’s retracted status in the References list and also include a citation and full reference for the retraction notice.

Additional Editor Comments (if provided):

[Note: HTML markup is below. Please do not edit.]

Reviewers' comments:

Reviewer's Responses to Questions

Comments to the Author

1. If the authors have adequately addressed your comments raised in a previous round of review and you feel that this manuscript is now acceptable for publication, you may indicate that here to bypass the “Comments to the Author” section, enter your conflict of interest statement in the “Confidential to Editor” section, and submit your "Accept" recommendation.

Reviewer #3: All comments have been addressed

Reviewer #4: (No Response)

________________________________________

2. Is the manuscript technically sound, and do the data support the conclusions?

The manuscript must describe a technically sound piece of scientific research with data that supports the conclusions. Experiments must have been conducted rigorously, with appropriate controls, replication, and sample sizes. The conclusions must be drawn appropriately based on the data presented.

Reviewer #3: Yes

Reviewer #4: Yes

________________________________________

3. Has the statistical analysis been performed appropriately and rigorously?

Reviewer #3: Yes

Reviewer #4: Yes

________________________________________

4. Have the authors made all data underlying the findings in their manuscript fully available?

The PLOS Data policy requires authors to make all data underlying the findings described in their manuscript fully available without restriction, with rare exception (please refer to the Data Availability Statement in the manuscript PDF file). The data should be provided as part of the manuscript or its supporting information, or deposited to a public repository. For example, in addition to summary statistics, the data points behind means, medians and variance measures should be available. If there are restrictions on publicly sharing data—e.g. participant privacy or use of data from a third party—those must be specified.

Reviewer #3: Yes

Reviewer #4: Yes

________________________________________

5. Is the manuscript presented in an intelligible fashion and written in standard English?

PLOS ONE does not copyedit accepted manuscripts, so the language in submitted articles must be clear, correct, and unambiguous. Any typographical or grammatical errors should be corrected at revision, so please note any specific errors here.

Reviewer #3: Yes

Reviewer #4: Yes

________________________________________

6. Review Comments to the Author

Please use the space provided to explain your answers to the questions above. You may also include additional comments for the author, including concerns about dual publication, research ethics, or publication ethics. (Please upload your review as an attachment if it exceeds 20,000 characters)

Reviewer #3: The authors attempt to identify a correlation between patient motivation and aerobic exercise capacity, specifically VO2 peak at baseline and change at the end of the cardiac rehabilitation program. It is well structured, novel, and written in good english. All the concerns addressed by the previous round of reviewers have been addressed adequately. Admittedly, the subgroup used for analysis restricts extrapolating this data to a younger age bracket, unfortunately in a male dominant population. Nevertheless, I do believe that the manuscript is of sufficient quality to warrant publication.

Comment

Reviewer #4: Interesting study about a clinically elevant topic. Impaired adherence of CR programs is one of the major issues when planning this kind of health resources. Describing predictors of success is very important. We know that CR adherence is low, even in randomized trials. I suggest to cite

Sanchis J, Sastre C, Ruescas A, Ruiz V, Valero E, Bonanad C, García-Blas S, Fernández-Cisnal A, González J, Miñana G, Núñez J. Randomized Comparison of Exercise Intervention Versus Usual Care in Older Adult Patients with Frailty After Acute Myocardial Infarction. Am J Med. 2021 Mar;134(3):383-390.e2. doi: 10.1016/j.amjmed.2020.09.019.

Answer:

Thank you for providing this interesting reference. It is indeed what we see as well in our clinic and we have sited Sanchis et al in the “Introduction” section on page 3.

I have some minor comments to the authors

Comment:

-Please explain the cutt-of of age. In this sense, 64 years is a very young cut off to be designated as elderly

Answer:

Thank you for raising this question. We agree that in many patients a cut-off of 65 is relatively low, however, there is considerable biological variation. Age 65 is the age of retirement in many European countries. Most importantly, however, is that the cut-of age of more than 64 years was decided in the EU-CaRE study group, which this paper derived data from. As EU-CaRE was a multi-country program, the 8 different countries had to agree of an acceptable cut-of age that all countries could accept. The definition of elderly is subject to discussion and we appreciate your relevant comment.

Comment:

-When talking about patients at older ages, frailty, comorbidity and other geriatric syndromes are closely related to prognosis, to the need for CR and probably to the motivation for CR. I understand the authors do not have information about geriatric assessment. In this case, this should be included as a limitation of this study

Answer: Thank you for this comment. We have added this to our limitations as we did not have a direct geriatric assessment. However, all patients did consult a cardiologist before CR who assessed if the patient were capable, also mentally, to complete a CR program.

Comment:

-Finally the authos should clearly discuss the clinical implications of their findings. Which is the recommendation for patients with low motivation values?. Probably these are the patients which higher need for CR...

Answer:

This is an interesting point and we have elaborated on the implications on page 9 under discussion and “Motivation as a predictor of VO_2peak_ before and after CR”

________________________________________

7. PLOS authors have the option to publish the peer review history of their article (what does this mean?). If published, this will include your full peer review and any attached files.

If you choose “no”, your identity will remain anonymous but your review may still be made public.

Do you want your identity to be public for this peer review? For information about this choice, including consent withdrawal, please see our Privacy Policy.

Reviewer #3: Yes: Dr Mark Abela MD (Melit) MRCP (London) MSc Internal Medicine (Edinburgh) MSc Sports Cardiology (London)

Reviewer #4: No

________________________________________

[NOTE: If reviewer comments were submitted as an attachment file, they will be attached to this email and accessible via the submission site. Please log into your account, locate the manuscript record, and check for the action link "View Attachments". If this link does not appear, there are no attachment files.]

While revising your submission, please upload your figure files to the Preflight Analysis and Conversion Engine (PACE) digital diagnostic tool, https://pacev2.apexcovantage.com/. PACE helps ensure that figures meet PLOS requirements. To use PACE, you must first register as a user. Registration is free. Then, login and navigate to the UPLOAD tab, where you will find detailed instructions on how to use the tool. If you encounter any issues or have any questions when using PACE, please email PLOS at figures@plos.org. Please note that Supporting Information files do not need this step.

________________________________________

In compliance with data protection regulations, you may request that we remove your personal registration details at any time. (Remove my information/details). Please contact the publication office if you have any questions.
